# Supplementary material for: Genetic Analysis of HIBM Myopathy-Specific GNE V727M Hotspot Mutation Identifies a Novel COL6A3 Allied Gene Signature That Is Also Deregulated in Multiple Neuromuscular Diseases and Myopathies
Source: Genes (Basel). 2023 Feb 24;14(3):567. doi: 10.3390/genes14030567 (PMC10048522; doi:10.3390/genes14030567)
Supplement: Supplementary file 1 [file genes-14-00567-s001.zip › Sup Table S1_ Primer details.pdf]

Primer details

Cloning primers	Forwarding primer (5′-3′)	Reveres primer (5′-3′)
GNE	ATAAAAGCTTTAGGCCATGGAAACCTATGG	ATAAGGATCCCTAGTAGATCCTGCG
SDM primers	Forwarding primer (5′-3′)	Reveres primer (5′-3′)
GNE SDM _V727M	GTGCAGGACGTGGATATGGTGGTTTCGGATTTGTTG	CAACAAATCCGAAACCACCATATCCACGTCCTGCAC
qPCR primers	Forwarding primer (5′-3′)	Reveres primer (5′-3′)
GNRH2	CTCTTCCTTGAGCAGCCATG	TAAGGGCATTCTGGGGATCC
PCP2	GGATCAGGAGGAGAAGACGG	CTGTCACACGTTGGTCATCC
MSR1	AGGACACTGATAGCTGCTCC	ACTGCAAACACGAGGAGGTA
