# Supplementary figures and images for: Genetic Analysis of HIBM Myopathy-Specific GNE V727M Hotspot Mutation Identifies a Novel COL6A3 Allied Gene Signature That Is Also Deregulated in Multiple Neuromuscular Diseases and Myopathies
Source: Genes (Basel). 2023 Feb 24;14(3):567. doi: 10.3390/genes14030567 (PMC10048522; doi:10.3390/genes14030567)

## Slide 1
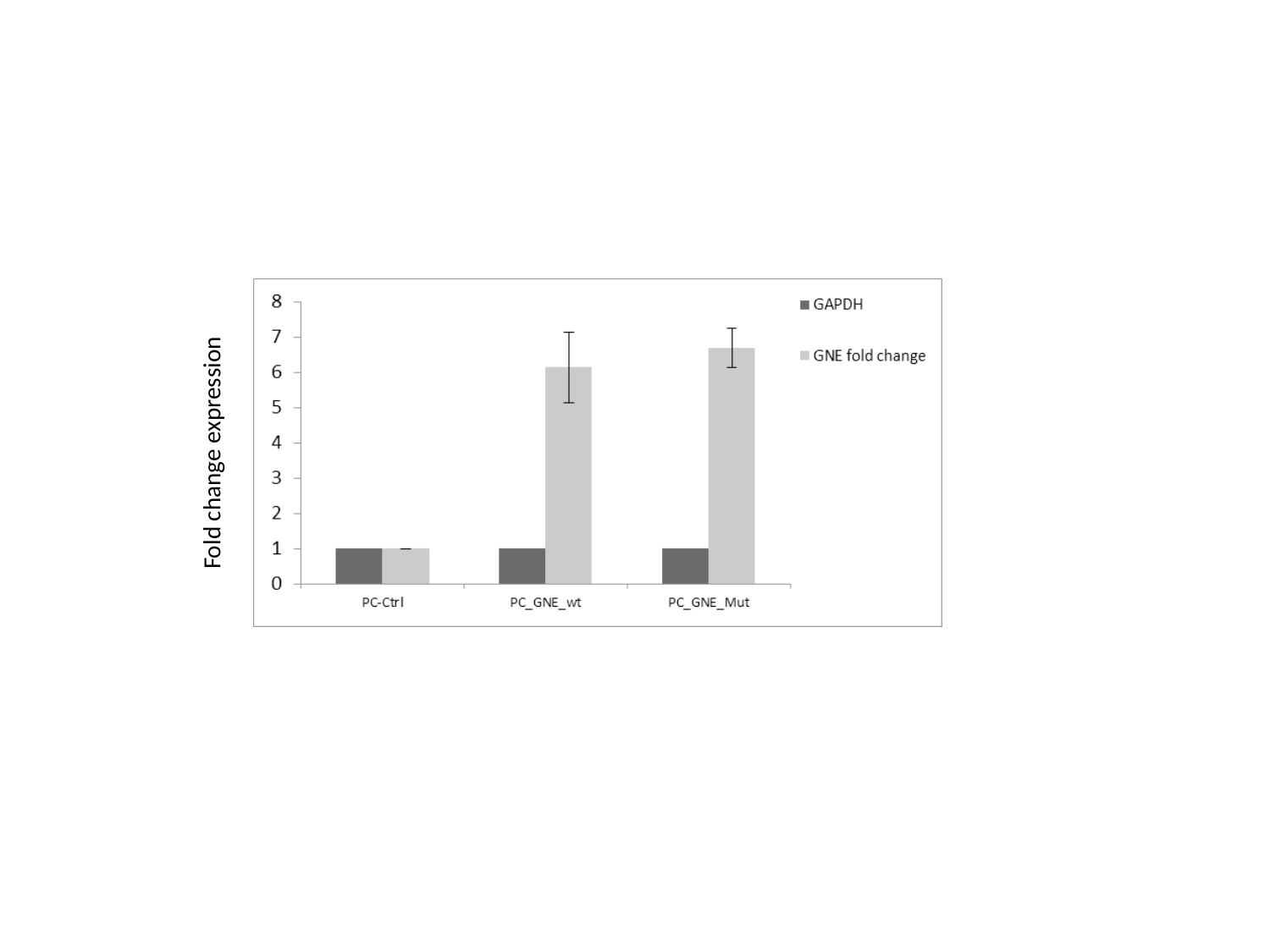

Fold change expression

Supplement: Supplementary file 1 [file genes-14-00567-s001.zip › Sup Figure S1.pptx]

## Slide 1
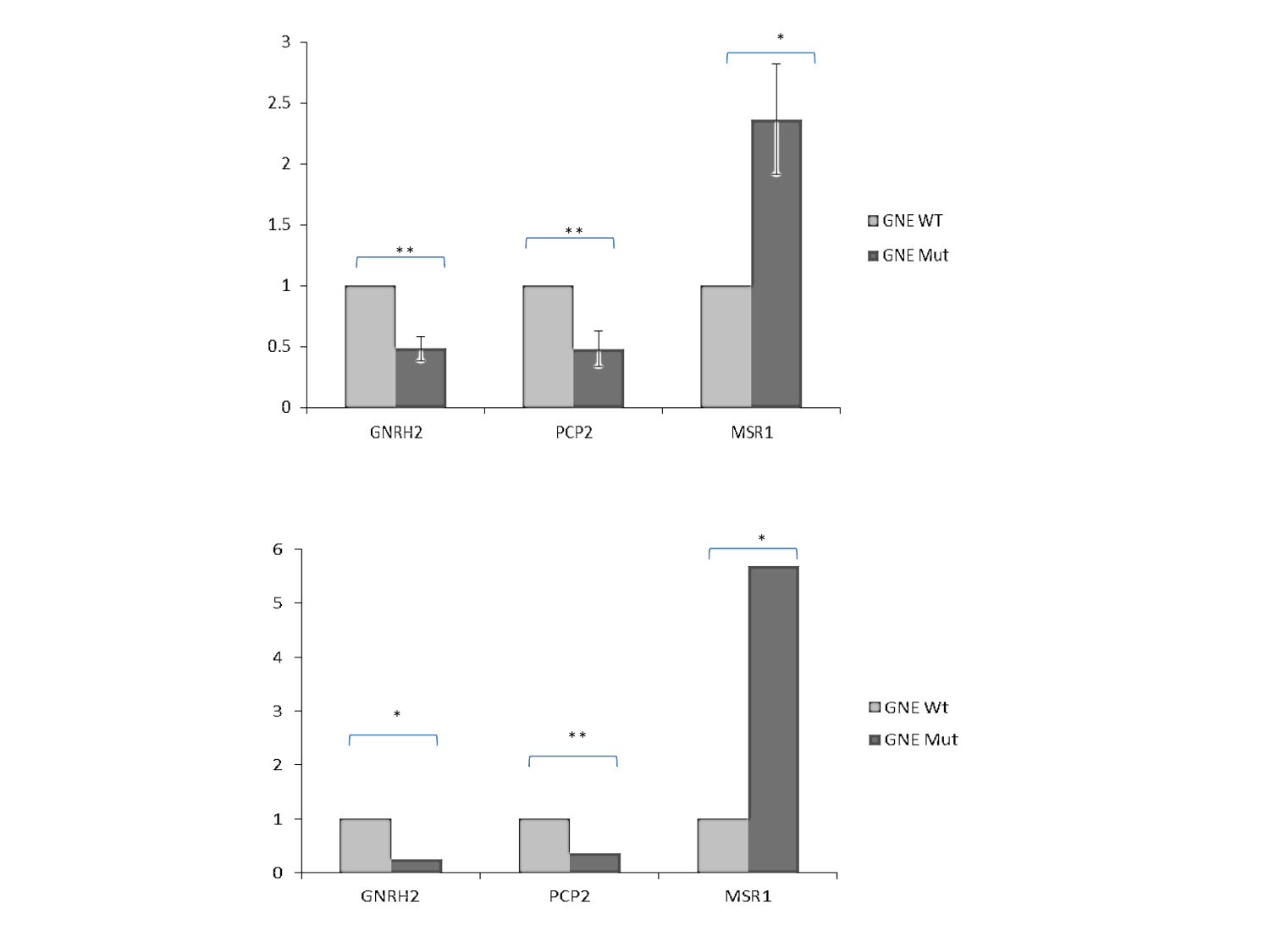

*
**
**
 *
 *
**

Supplement: Supplementary file 1 [file genes-14-00567-s001.zip › Sup Figure S2.pptx]
